# Improving the standards of scientific publishing in India

**DOI:** 10.4103/0971-5916.73393

**Published:** 2010-11

**Authors:** Naresh Kasoju, Utpal Bora

**Affiliations:** Biomaterials & Tissue Engineering Laboratory, Indian Institute of Technology Guwahati, Guwahati, Assam 781 039, India

## 

Sir,

Scientific publishing capabilities, both in terms of quality and quantity, to a great extent reflect the strength of science and technology of a country. Many developing countries, including India, are yet to achieve success in this aspect. In the recent past, the order of global leaders is on a transition and the “east” is now giving a tough competition to “west” in terms of scientific and technological power^1^. But, India is struggling for a superior position this competition. When it comes to number, India publishes a huge number of journals; there are about 600 journals in biomedical subject alone^2^. But, the quality of these journals remains largely ignored. In a recent editorial, some common problems encountered in scientific publishing in India, especially in biomedical subject are discussed with great concern^2^. It also described the role of journal core team (including editor and other editorial team) in bringing up these journals in the forefront of global community^2^. Here we share few other concerns which if properly addressed, could contribute to the success of scientific publishing in India.

### 1. Funding agencies

The prestigious funding bodies under the Government of India, such as Department of Science & Technology, Department of Biotechnology, Indian Council of Medical Research, Council of Scientific & Industrial Research, *etc*., should recommend the researchers to publish their work preferably in relevant Indian journals. The funding bodies should announce a special package/reward/prize, in some form or the other, for researchers who publish their research in Indian journals.

### 2. Research community

It is now a well accepted fact that the Asian scientific power is soaring at a rapid pace, and a sense of “look east” has developed in the minds of global community. But, unfortunately, the Indian research community (both scientists and students), though aware of the above fact, still believe in “west-first” approach to get a good impact factor (IF)^3^ publication. An exercise is required to change the mindset of researchers working in various laboratories (government funded labs, colleges, universities, autonomous institutes, *etc*.) and their preference should shift to “good-will factor” rather than “impact factor”.

### 3. Public sector

One cannot blame the scientific community for their “west-first” tendency to get high IF papers. The existing recruitment policies make them more focused on their personal career over national pride. For example, if a Ph.D. scholar, having publications in Indian journals (with zero/lower IF), applies for a position (either post-doc or any permanent/temporary job) in any institute, the chances of selection are slim. Even the scientists/ faculty with regular appointment also would like to improve their IF to get promoted to a higher grade. Hence, unless and until government makes a policy to regulate “IF” as a criteria for selection/promotion of a candidate, researchers are forced to plan work which has a potential to get a high IF rather than work for problems inflicting the nation. Only when amendments in recruitment criteria are made, the researchers would try to balance between “good-will factor” and “impact factor” and thereby would be able to balance “personal career” and “national pride”.

The government should initiate an agency/body to develop and maintain a single window web portal for hosting all Indian journals. The best example of such kind is J-STAGE (Japan Science and Technology Information Aggregator, Electronic) developed and maintained by JST (Japan Science and Technology) Agency of Government of Japan. J-STAGE is a huge collection of research articles reflecting the science and technology strength of Japan. Currently, it includes 639 journals (313, 237 articles), 125 proceedings, 10 reports and 42 JST reports (as accessed on August 31, 2010)^4^.

The government should fund the publishers or societies (which have their official publications) for “open access” publication. Consequently, the research published in Indian journals reach every corner of the globe free of cost, and hence, attracts the global scientific community to Indian journals.

### 4. Private sector

There is a tremendous scope for private sector groups in scientific publishing in India. With the help of indigenous IT sector, private venture capitalists should take initiations towards launch of a single window portal for all Indian journals including providing access to full text, searching options, submission and peer-review, *etc*. The best examples are websites like NPG, Elsevier, Wiley, Springer, *etc*., which provide such facilities.

### 5. Journal team

Besides the solutions suggested in a recent editorial by Satyanarayan & Sharma^2^, in our opinion, following are few more issues a journal’s editorial team should consider, to improve the standards:

- The journals should adapt to the regular changes happening in their fields and should think of expanding the aim and scope as per the demands of scientific community.
- On a regular basis, the journal team should send circular/poster inviting submissions to various research institutes (Indian and foreign). Again ethically, this should be an unbiased invitation.
- The journals should put more efforts in making a user-friendly web based system for accessing full text content, searching, submission and review. For this, the journals can attract funds by hosting third party advertisements.
- Journals should provide templates for various forms of articles that they publish. This will help the researchers to a great extent, especially those in the early stages of their career.
- Global outreach and impact factor is all what a researcher expects. So, editorial team should seriously think on this issue.
- Last but not the least, a journal should encourage the authors to give citations to work published in that particular journal. It is not a good option, but not a bad one too! Many journals belonging to NPG, Elsevier, *etc*., encourage such self-citations.

China, Japan and Korea are collaborating with agencies like NPG, Elsevier, *etc*. to bring their journals in the forefront of global community and thereby improving their standards. Recent trends in science and technology all over the world clearly indicating that China is on rise at a rapid pace^5^. Though the data show that India is also moving on in this direction, the target is still miles ahead^6^. It is time to think. It is really the time for “publish in India” movement^7^. We strongly believe that a collective effort from funding agencies, researchers, public and private sectors, and journals’ editorial teams, would certainly transform India as the global leader in scientific publishing.

## References

[1] Anon (2007). Asian on the rise. *Nature*.

[2] Satyanarayana K, Sharma A (2010). Biomedical Journals in India: Some critical concerns. *Indian J Med Res*.

[3] Satyanarayana K, Sharma A (2008). Impact factor: Time to move on. *Indian J Med Res*.

[4] http://www.jstage.jst.go.jsp/browse.

[5] Satyanarayana K (2010). India, China, and the world. *Indian J Med Res*.

[6] Satyanarayana K (2007). We are surging ahead!. *Indian J Med Res*.

[7] Satyanarayana K (2004). Time for ‘Publish in India’ movement. *Indian J Med Res*.

